# A novel CT-based radiomics in the distinction of severity of coronavirus disease 2019 (COVID-19) pneumonia

**DOI:** 10.1186/s12879-021-06331-0

**Published:** 2021-06-25

**Authors:** Zongyu Xie, Haitao Sun, Jian Wang, He Xu, Shuhua Li, Cancan Zhao, Yuqing Gao, Xiaolei Wang, Tongtong Zhao, Shaofeng Duan, Chunhong Hu, Weiqun Ao

**Affiliations:** 1 Department of Radiology, The First Affiliated Hospital of Bengbu Medical College, No. 287 Changhuai Road, Bengbu, 233004 Anhui China; 2 Shanghai Institute of Medical Imaging, and Department of Interventional Radiology, Zhongshan Hospital, Fudan University, No. 180 Fenglin Road, Xuhui District, Shanghai, 200032 China; 3 Department of Radiology, Tongde Hospital of Zhejiang Province, No.234, Gucui Road, Hangzhou, 310012 Zhejiang Province China; 4 Department of Radiology, Fuyang Second People’s Hospital, No. 450 Linquan Road, Fuyang, 236000 Anhui China; 5 GE Healthcare China, Pudong new town, No1, Huatuo road, Shanghai, 210000 China; 6 Department of Radiology, The First Affiliated Hospital of Soochow University, No. 188, Street of Shizi, Suzhou, 200000 China

**Keywords:** COVID-19, Radiomics, Tomography, X-ray computed, Nomogram

## Abstract

**Background:**

Convenient and precise assessment of the severity in coronavirus disease 2019 (COVID-19) contributes to the timely patient treatment and prognosis improvement. We aimed to evaluate the ability of CT-based radiomics nomogram in discriminating the severity of patients with COVID-19 Pneumonia.

**Methods:**

A total of 150 patients (training cohort *n* = 105; test cohort *n* = 45) with COVID-19 confirmed by reverse transcription polymerase chain reaction (RT-PCR) test were enrolled. Two feature selection methods, Max-Relevance and Min-Redundancy (mRMR) and least absolute shrinkage and selection operator (LASSO), were used to extract features from CT images and construct model. A total of 30 radiomic features were finally retained. Rad-score was calculated by summing the selected features weighted by their coefficients. The radiomics nomogram incorporating clinical-radiological features was eventually constructed by multivariate regression analysis. Nomogram, calibration, and decision-curve analysis were all assessed.

**Results:**

In both cohorts, 40 patients with COVID-19 pneumonia were severe and 110 patients were non-severe. By combining the 30 radiomic features extracted from CT images, the radiomics signature showed high discrimination between severe and non-severe patients in the training set [Area Under the Curve (AUC), 0.857; 95% confidence interval (CI), 0.775–0.918] and the test set (AUC, 0.867; 95% CI, 0.732–949). The final combined model that integrated age, comorbidity, CT scores, number of lesions, ground glass opacity (GGO) with consolidation, and radiomics signature, improved the AUC to 0.952 in the training cohort and 0.98 in the test cohort. The nomogram based on the combined model similarly exhibited excellent discrimination performance in both training and test cohorts.

**Conclusions:**

The developed model based on a radiomics signature derived from CT images can be a reliable marker for discriminating the severity of COVID-19 pneumonia.

## Background

Coronavirus disease 2019 (COVID-19) has become a global pandemic since it started in December 2019 [1]. Although most of the confirmed patients with COVID-19 are mild, about 20% cases can still be severe [1]. Pneumonia can be developed in COVID-19 patients [2] . In some severe patients with COVID-19, dyspnea was observed more than 1 week after the onset of symptoms [3]. Septic shock, acute respiratory distress syndrome, difficulty in correction of metabolic acidosis, and coagulation dysfunction are often developed rapidly in severe patients [4]. The critical factor for decreasing complication and mortality is the effective diagnosis of severe patients. In other words, convenient and precise assessment of the severity in COVID-19 will contribute to the timely patient treatment and prognosis improvement.

CT examination can be severed as an important assistant tool for diagnosing COVID-19 [4–7]. As the literature reveals [4, 5], the sensitivity of imaging examination, especially CT imaging, is relatively high, and the imaging signs can manifest earlier than the clinical symptoms, thus CT examination is significant in preclinical screening, primary diagnosis, and evaluation of disease severity. Although recent studies have reported CT findings of the COVID-19 pneumonia [5, 6], the value of CT imaging in assessing the severity of the patients with COVID-19 were scarcely reported, which, however, may be more conducive to our in-depth comprehensive and accurate understanding of this new infectious disease. Radiomics, as an emerging technique involved with the extraction of high-throughput data from quantitative imaging features and the subsequent association of this parameter with clinical data, has been applied in various diseases. For example, radiomics have often been applied in discrimination of tumors and prediction of histologic grade, tumor recurrence and metastasis [7, 8]. Presumably, CT-based radiomics has great advantage in the diagnosis and follow-up of COVID-19 pneumonia. As far as we know, the existing literature mainly focused on identification and diagnosis of COVID-19 [9, 10]. Some studies [11–13] have identified CT-based radiomics as a superior tool for discriminating COVID-19 and other types of viral pneumonia or non-COVID-19 pneumonia. Few literature has reported the application of CT-based radiomics for evaluation of the severity of COVID-19.

Therefore, the purpose of this study was to apply the CT-based radiomics nomogram, combining radiomics signatures and clinical factors, for the discrimination of the severity of COVID-19 pneumonia, helping to optimize therapeutic regiment.

## Methods

### Demographic data

Between January and February 2020, a total of 213 patients from 2 hospitals in Anhui, China diagnosed as COVID-19 pneumonia were enrolled. This retrospective study was approved by the Ethics of Committees of the First Affiliated Hospital of Bengbu Medical College and informed consent for this retrospective study was waived. All of the procedures were performed in accordance with the Declaration of Helsinki in 1964 and relevant policies in China.

Our inclusion criteria were: (a) confirmed positive by real-time reverse-transcriptase polymerase-chain-reaction (RT-PCR) assay from nasal and pharyngeal swab specimens; (b) scanned with thin-section CT; (c) CT images demonstrated pneumonia; (d) CT examination was done at the patient’s first visit. Exclusion criteria were as follows: (a) lack of complete medical data (*n* = 31); (b) patients without thin-section CT or lack of CT images (*n* = 32). Finally, 150 patients were collected and divided into severe group and non-severe group. According to the guideline of American Thoracic Society Criteria, the severe patient was defined as meeting any of the following conditions [14]: a) respiratory rate ≥ 30 breaths/min; b) respiratory distress; c) finger oxygen saturation ≤ 93% in resting state; d) arteria oxygen tension (PaO2)/inspiratory oxygen fraction (FiO2) ≤ 300 mmHg; e) mechanical ventilation required and respiratory failure occurred; f) the presence of shock; g) patients with other organ failures required ICU monitoring and treatment. The complete medical data including demographic characteristics, epidemiological information, laboratory data, symptoms, comorbidity, and medical treatment data were recorded.

### CT acquisition

Patients underwent chest CT imaging on two 64-detector CT scanners (LightSpeed, GE and Aquilion, TOSHIBA). The protocols were as follows: 120 kV; automatic tube current (350 mA for LightSpeed, GE and 440mAs for Aquilion, TOSHIBA); detector width, 40 mm (Light Speed, GE) and 43 mm (Aquilion, TOSHIBA); rotation time, 0.8 s (Light Speed, GE) and 1.0 s (Aquilion, TOSHIBA); section thickness, 5 mm; interlayer spacing, 5 mm; matrix, 512 × 512; and breath hold at full inspiration. The following windows were used for image display: a mediastinal window with window width of 350 HU and window level of 40 HU and a lung window with a width of 1200 HU and window level of − 600 HU. The acquired images were subsequently reconstructed using iterative reconstruction technique with a slice thickness of 0.625 mm (LightSpeed, GE) and 1.25 mm (Aquilion, TOSHIBA), respectively. To minimize discrepancies in the image acquisition parameters attributable to the different CT machines, all images were resampled to a 1 × 1 × 1 mm^3^ voxel size before image analysis and feature extraction.

### Clinical findings and laboratory tests

The confirmed COVID-19 patients must be treated in isolation. Patients underwent symptomatic treatment, including electrolyte turbulence correction, anti-infection treatment, nutritional support and bed rest. When the patient was perceived to have difficulty in breathing, they were placed to the ventilator.

The time course (defined as the interval between the onset of symptoms and the initial CT examination), main clinical features (age, gender, fever and cough), comorbidity (such as diabetes, hypertension, chronic liver disease, cardiac disease and chronic obstructive pulmonary disease) and main laboratory tests (C-reactive protein; lymphocyte count, and blood leukocyte count) were independently reviewed by two clinicians.

### Image interpretation

Main CT signs were analyzed as follows: pure ground glass opacity (GGO), GGO with consolidation; consolidation; interlobular septal thickening; crazy-paving pattern; halo sign; reversed halo sign; air bronchogram; pleural effusion. Lesion distribution was described as left, right or bilateral lungs. The CT scoring was based on the involvement of the lung segment using 18 lung segments model from 1 to 4: 1, 1–4 lung segments involvement; 2, 5–8 lung segments involvement; 3, 9–12 lung segments involvement; 4, 13–18 lung segments involvement. Number of lesions was defined by reference to previous literature [15]. For instance, it is counted as one when the lesion only occupies one lung segment. When a large lesion involves more than one lung segment, it is counted as the number of affected lung segments. All radiological data were independently reviewed by two radiologists (with 6 and 13 years of experience in chest CT imaging, respectively). The radiologists were blinded to the clinical data of all the patients. If there was a disagreement, a third observer (with 18 years of experience in chest CT imaging) was asked for an opinion and a majority decision was reached.

### CT images segmentation and features extraction

Two radiologists (R1, 6 years’ and R2, 13 years’ experience in chest imaging) segmented lesions using ITK-SNAP software. A 2D region of interest (ROI) was used to delineate lesions in coronal slice with the largest section of lesions. In order to improve model robustness, one radiologist segmented lesions 2 times with a time interval of a week, and the other one segmented once.

The images were firstly preprocessed using resampling, intensity discretion methods. All images were resampled into 1 × 1 × 1 mm^3^ of voxel size. Resampling aimed to transform the image into the isotropic voxel spacing to ensure the texture features were rotationally invariant and comparable between the images coming from different scanners. The intensity discretion was conducted to change the gray-level into 128 bins to reduce the complexity of calculation and make features tractable.

We used AK software (Artificial intelligence Kit, GE Healthcare) to extract the radiomic features based on the preprocessed image. We finally got three classes of features: the histogram features, texture features (355, based on GLCM, RLM, GLSZM) and geometry features. A total of 396 radiomic features were extracted. In order to construct a robust model, the inter-observer agreement and intra-observer agreement tests were performed to acquire the reproducible features. Each lesion has 3 ROIs, 2 ROIs from R1, 1 from R2. The features extracted based on the ROIs of R1 were used to test the intra-observer agreement, meanwhile, the first ROI of R1 and the ROI of R2 were acquired to test the inter-observer agreement. The feature that met the two tests were retained for constructing the diagnosis model and the R1’s first ROI was adopted [8].

### Radiomics model construction

The cohorts were grouped into training cohort and test cohort using stratified random resampling method with a ratio of 7:3. Unless the emphasis of using the validation cohort, the following operation were all performed in training cohort. Before constructing the radiomic model, the feature engineering was conducted. Three feature selection steps were adopted. The first step was to exclude the zero-variance features. Zero-variance meant the values of the feature were same across training cohort, and couldn’t be used for discrimination. The second step was using mRMR method to exclude the redundant features and kept the most relevant features with targets. After mRMR, 30 features were retained. The last step was using LASSO regression method to find the most predictive feature subset, which included two steps. We initially determined the optimized hyperparameter λ using 10-fold cross validation with binomial deviance as criterion. After the λ was determined, the features with non-zero coefficient were the last chosen features. LASSO regression was conducted to construct the radiomics model, which also meant to get the Rad-score. The Rad-score could be calculated by summing the features multiplied their corresponding coefficients.

### Clinical and morphologic risk features

In addition to the radiomic features, we also collected the radiological features and clinical data (collectively named “clinical feature” later). The clinical feature was used to construct the clinical model. Firstly, we used two samples’ or κ^2^ test to assess whether the clinical features were significantly different between two groups. The significantly different features were subsequently analyzed using the univariate logistic regression, the features with non-zero coefficients in univariate logistic regression analysis were integrated into backward stepwise selection multivariable logistic regression analysis [16]. Meanwhile, the clinical model was built.

### Nomogram construction

After the Rad-score was calculated and clinical factors were selected using backward step-wise multivariate logistic regression, the Rad-score and the remaining clinical features were subsequently combined to construct the nomogram using multivariate logistic regression.

### Model validation

**Fig. 1:**
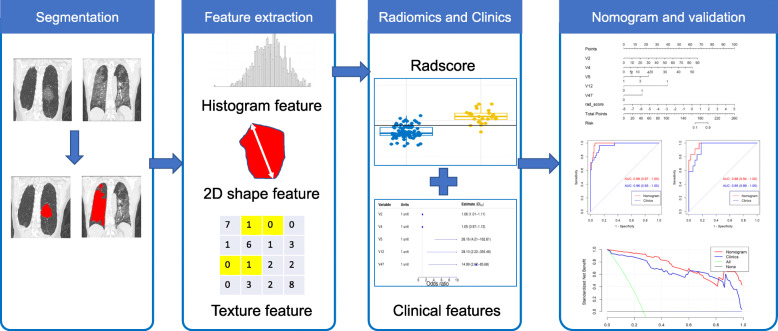
Flowchart of radiomics procedure in this study

The receiver operating characteristic (ROC) analysis was performed to evaluate the performance of the radiomic model, clinical model and nomogram. Accuracy, sensitivity, specificity, positive predictive value (PPV) and negative predictive value (NPV) were obtained from the cohorts. Besides, the calibration curves were plotted to assess the agreement between the predicted event probability and observed event probability, and Hosmer-Lemeshow statistic was applied to test the difference between the predicted event probability and observed event probability. Decision curve analysis (DCA) was finally utilized to determine the clinical utilities. The flowchart of segmentation, feature extraction, and model building is depicted in Fig. 1.

### Statistical analysis

The statistical analyses were all performed using R software (version 3.6.1, R Core Team (2019). R: A language and environment for statistical computing. R Foundation for Statistical Computing, Vienna, Austria. URL www.r-project.rog). The inter-observer and intra-observer agreement tests were performed using ‘DescTools’ package to calculate the intraclass correlation coefficient (ICC). The features with ICC > 0.75 was treated as reproducibility (7). The ‘caret’ package was used to split the cohort, preprocess the features, build the confusion matrix to get the accuracy, sensitivity, specificity, PPV and NPV. mRMR feature selection was conducted using the ‘mRMRe’ package. ‘pROC’ package was used to perform ROC analysis, while ‘ModelGood’ package and ‘rmda’ package was used to perform calibration curve analysis and decision curve analysis, respectively. Two-sided *p* <  0.05 indicated statistical significance.

## Results

### Clinical and radiological features

A total of 150 patients (68 men and 82 women) confirmed with COVID-19 was classified into a training cohort (*n* = 105, 28 were severe and 77 were non-severe; 43 men and 62 women) and a test cohort (*n* = 45, 12 were severe and 33 were non-severe; 25 men and 20 women). Comparisons of clinical features, laboratory, and CT image features in training and test cohorts are shown in Tables 1 and 2, respectively.

**Table 1:** Comparison of severity and clinical characteristics in both training and test cohort

Clinical characteristics	Training cohort		*p* value	Test cohort		*p* value	*p* value*
	Non-severe (*n* = 77)	Severe (*n* = 28)		Non-severe (*n* = 33)	Severe (*n* = 12)		
Age	43.4 ± 14.6	55.6 ± 12.6	< 0.001	43.4 ± 12.4	59.9 ± 19.8)	0.001	0.674
Gender							0.828
Male	34 (44.2)	9 (32.1)	0.377	16 (48.5)	9 (75.0)	0.214	
Female	43 (55.8)	19 (67.9)		17 (51.5)	3 (25.0)		
Fever			0.999 (86.2%)			0.269	0.439
Absence	9 (11.6%)	3 (10.7%)		5 (15.2%)	1 (8.3%)		
Presence	72 (86.7%)	22 (88.0%)		28 (84.8%)	11 (91.7%)		
Cough			0.054			0.999	0.521
Absence	43 (55.8)	9 (32.1)		14 (42.4)	5 (41.7)		
Presence	34 (44.2)	19 (67.9)		19 (57.6)	7 (58.3)		
C-reaction protein			0.447			0.448	0.853
Absence	15 (19.5)	3 (10.7)		8 (24.2)	1 (8.3)		
Presence	62 (80.5)	25 (89.3)		25 (75.8)	11 (91.7)		
Blood leukocyte count			0.088			0.999	0.805
< 4 × 10^9^/L	26 (33.8)	16 (57.1)		21 (63.6)	6 (50.0)		
(4–10) × 10^9^/L	50 (64.9)	12 (42.9)		12 (36.4)	6 (50.0)		
> 10 × 10^9^/L	1 (1.3)	0 (0.0)		0 (0.0)	0 (0.0)		
Lymphocyte count (< 1.5 × 10^9^/L)	31 (40.3)	7 (25.0)	0.227	13 (39.4)	1 (8.3)	0.104	0.680
Comorbidity			< 0.001			0.228	0.103
Absence	61 (79.2)	10 (35.7)		29 (87.9)	8 (66.7)		
Presence	16 (20.8)	18 (64.3)		4 (12.1)	4 (33.3)		
Time course (day)	6.8 ± 3.8	9.0 ± 4.9	0.016	6.8 ± 4.0	5.5 ± 3.9	0.345	0.209

*Represents the comparisons of features between training and test cohorts

**Table 2:** Comparison of severity and radiological characteristics in both training and test cohort

radiological characteristics	Training cohort		*p* value	Test cohort		*p* value	*p* value*
	Non-severe (*n* = 77)	Severe (*n* = 28)		Non-severe (*n* = 33)	Severe (*n* = 12)		
Number of lesions	11.7 ± 7.9	21.8 ± 10.1	< 0.001	11.6 ± 8.5	18.6 ± 6.6	0.009	0.576
CT score			< 0.001			< 0.001	0.982
1	20 (26.0)	0 (0.0)		9 (27.3)	0 (0.0)		
2	20 (26.0)	0 (0.0)		8 (24.2)	0 (0.0)		
3	21 (27.3)	2 (7.1)		10 (30.3)	1 (8.3)		
4	16 (20.8)	26 (92.9)		6 (18.2)	11 (91.7)		
Distribution of lesions			0.085			0.450	0.767
Left lung	4 (5.2)	0 (0.0)		2 (6.1)	0 (0.0)		
Right lung	8 (10.4)	0 (0.0)		2 (6.1)	0 (0.0)		
Both lungs	65 (84.4)	28 (100.0)		29 (87.9)	12 (100.0)		
Pure GGO			0.445			0.669	0.999
Absence	8 (13.4)	5 (17.9)		4 (13.2)	2 (16.7)		
Presence	69 (89.6)	23 (82.1)		29 (87.8)	10 (83.3)		
Consolidation			0.213			0.204	0.999
Absence	61 (79.2)	28 (100.0)		26 (78.8)	12 (100.0)		
Presence	16 (20.8)	0 (0.0)		7 (21.2)	0 (0.0)		
GGO with consolidation			0.013			0.027	0.482
Absence	22 (28.6)	1 (3.6)		13 (39.4)	0 (0.0)		
Presence	55 (71.4)	27 (96.4)		20 (60.6)	12 (100.0)		
Crazy-paving pattern			0.005			0.344	0.089
Absence	36 (46.8)	4 (14.3)		9 (27.3)	1 (8.3)		
Presence	41 (53.2)	24 (85.7)		24 (72.7)	11 (91.7)		
Halo sign			0.012			0.228	0.823
Absence	11 (14.3)	11 (39.3)		4 (12.1)	4 (33.3)		
Presence	66 (85.7)	17 (60.7)		29 (87.9)	8 (66.7)		
Reversed halo sign			0.861			0.576	0.999
Absence	63 (81.8)	24 (85.7)		26 (78.8)	11 (91.7)		
Presence	14 (18.2)	4 (14.3)		7 (21.2)	1 (8.3)		
Interlobular septal thickening			0.072			0.109	0.148
Absence	30 (39.0)	5 (17.9)		9 (27.3)	0 (0.0)		
Presence	47 (61.0)	23 (82.1)		24 (72.7)	12 (100.0)		
Air bronchogram			0.087			0.344	0.546
Absence	26 (33.8)	4 (14.3)		9 (27.3)	1 (8.3)		
Presence	51 (66.2)	24 (85.7)		24 (72.7)	11 (91.7)		
Pleura effusion			0.999			0.593	0.999
Absence	74 (96.1)	27 (96.4)		33 (100.0)	11 (91.7)		
Presence	3 (3.9)	1 (3.6)		0 (0.0)	1 (8.3)		
Rad-score^a^	−3.5 (−4.2, −1.9)	1.2 (0.3, 2.1)	< 0.001	−2.9 [−3.7, −1.7]	−0.1 (−1.2, 1.5)	< 0.001	0.613

*Represents the comparisons of features between training and test cohorts

^a^ Data are showed as medians (IQR 25–75)

**Fig. 2:**
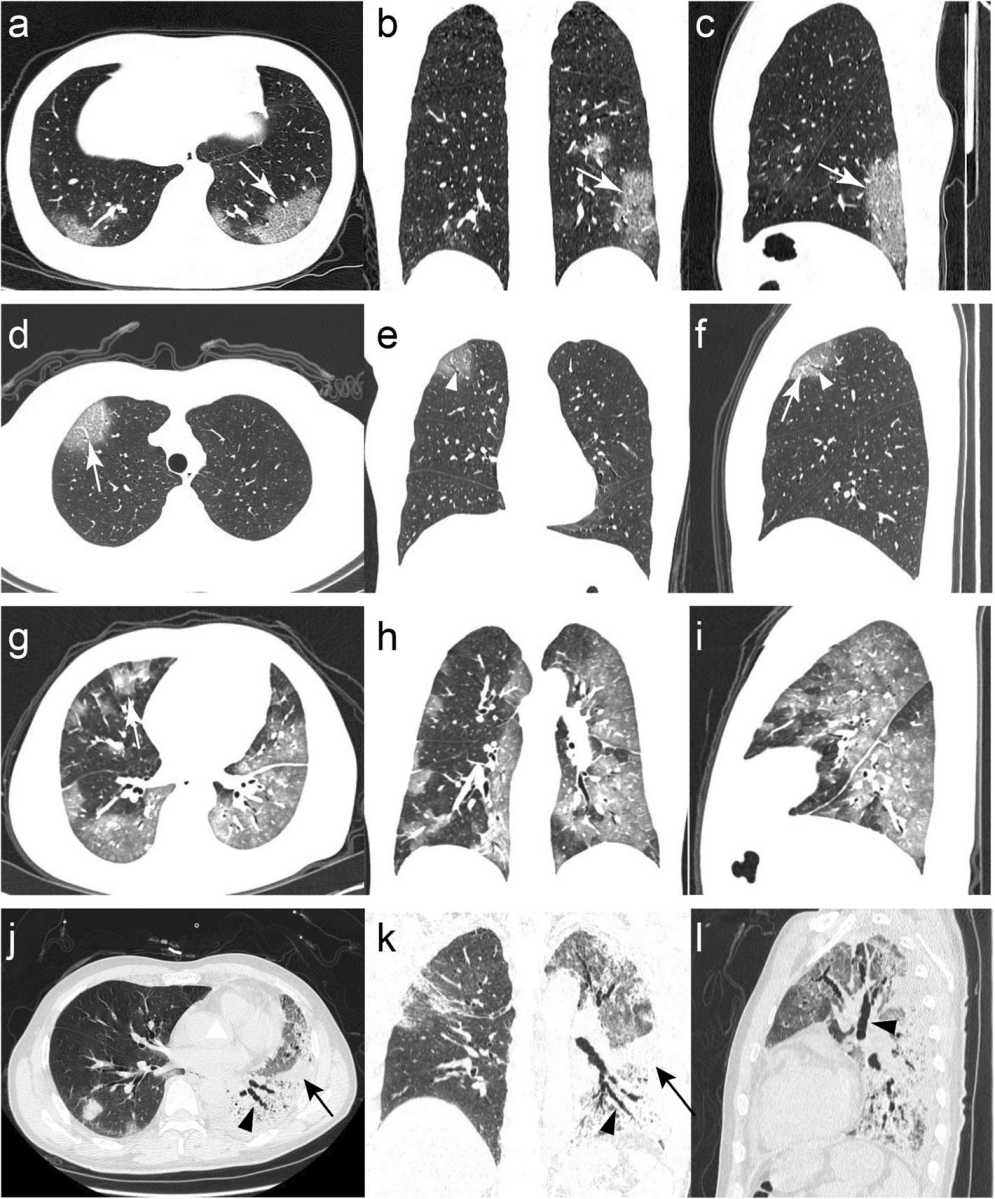
Thin-section CT images for severe and non-severe patients. **a**-**c** Images of a 25-year-old woman with non-severe COVID-19 pneumonia (CT score = 2) who had the symptoms of dry cough and fever. The axial, coronal and sagittal CT images all presented subpleural GGO (with craving stone sign) in the lower lobes of both lungs (white arrows). **d**-**f** Images of a 55-year-old woman with non-severe COVID-19 pneumonia (CT score = 1) who had the symptom of fever. The axial, coronal and sagittal CT images all presented GGO in the anterior segment of the upper lobe of the right lung, containing air bronchogram (white arrowheads) and vascular thickening (white arrow). **g**-**i** Images of a 52-year-old man with severe COVID-19 pneumonia (CT score = 4) who had the features of fever and comorbidity (diabetes, hypertension). The axial, coronal and sagittal CT images showed diffuse large regions of GGO with partial consolidation and interlobular septal thickening (white arrow). **j**-**l** Images of a 64-year-old man with severe COVID-19 pneumonia (CT score = 4) who had the symptoms of fever and cough. The axial, coronal and sagittal CT images showed diffuse large regions of GGO, accompanying consolidation (black arrows), and beaded air bronchogram (black arrowheads)

Of the 150 COVID-19 patients, 40 (26.7%) were severe and 110 (73.3%) were non-severe (Fig. 2). All parameters between the training and test datasets showed no statistically significant difference. By multivariable analysis, we found age [odds ratio (OR):1.073; 95% CI: 1.001–1.172; *p* = 0.069)], number of lesions (OR:1.100; 95% CI: 1.005–10,239; *p* = 0.066), CT scores (OR: 13.223; 95% CI: 2.718–141.341; *p* = 0.008), GGO with consolidation (OR: 31.084; 95% CI: 0.272–1033.61; *P* = 0.018), and comorbidity (OR: 20.104; 95% CI: 3.765–183.208; *p* = 0.002) were significant independent predictors of severity of COVID-19 patients (Table 3). The ROC curves were performed in the training cohort and test cohort, whose AUCs were 0.96 (0.93–1.00) and 0.95 (0.89–1.00), respectively. The accuracy, sensitivity, specificity, PPV and NPV were 0.89, 0.96, 0.87, 0.73, 0.95 in the training cohort, while they were 0.87, 0.73, 0.95, 0.83, 0.88, 0.71 in the test cohort.

**Table 3:** Univariate and Multivariate Analyses of Factors for assessing severity of COVID-19 Pneumonia

Risk factors	Univariate Analysis			Multivariate Analysis		
	OR	95%CI	*P* value	OR	95%CI	*P* value*
Age	1.072	1.033–1.12	0.001	1.073	1.001–1.172	0.069
Time course	1.126	1.018–1.254	0.024			
Number of lesions	1.134	1.072–1.215	0.001	1.100	1.005–1.239	0.066
CT scores	19.222	5.715–123.482	0.001	13.223	2.718–141.341	0.008
GGO with consolidation	10.800	2.09–198.469	0.023	31.084	2.727–1033.61	0.018
Crazy-paving pattern	5.268	1.826–19.205	0.005			
Halo sign	0.258	0.094–0.694	0.007			
Interlobular septal thickening	2.936	1.073–9.488	0.049			
Cough	2.670	1.097–6.901	0.035			
Comorbidity	6.862	2.718–18.362	0.001	20.104	3.765–183.208	0.002

*OR* odds ratio, *CI* confidence interval

*Multivariable logistic regression analysis utilized backward stepwise selection and AIC as criterion

### Radiomics model

**Fig. 3:**
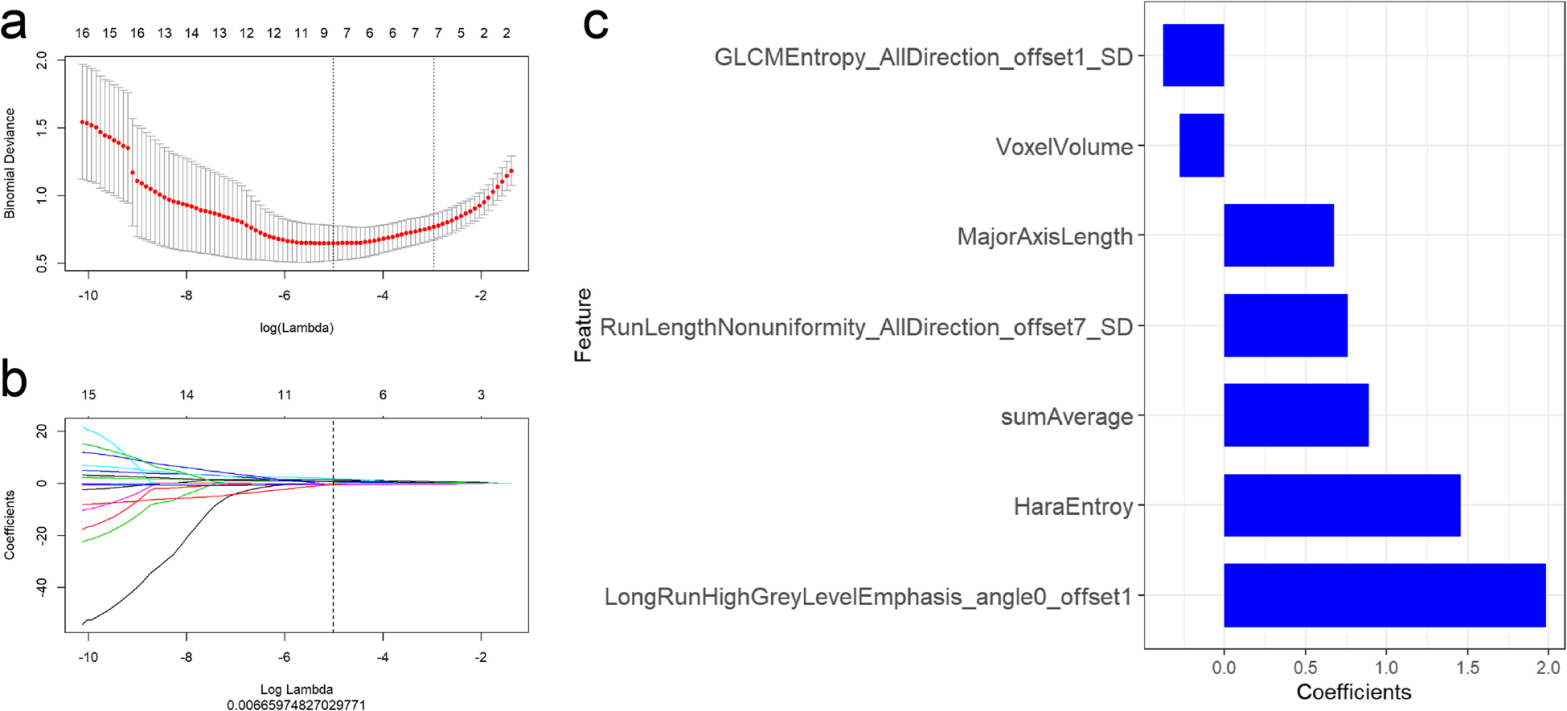
Feature selection via the least absolute shrinkage and selection operator (LASSO) binary logistic regression model. **a**The LASSO regression method was utilized to select radiomic features. A 10-fold cross-validation method was utilized to screen hyperparameter (λ) of the LASSO regression model and choose the model with the smallest error (λ), **b** LASSO coefficient profiles of the features represent vertical lines that are drawn at the value selected via 10-fold cross-validation, and the optimized hyperparameter λ was determined to be 0.00677, and 7 radiomic features were remained. **c** By LASSO logistic regression analysis, 7 optimal radiomic features were identified for reconstructing the prediction model

**Fig. 4:**
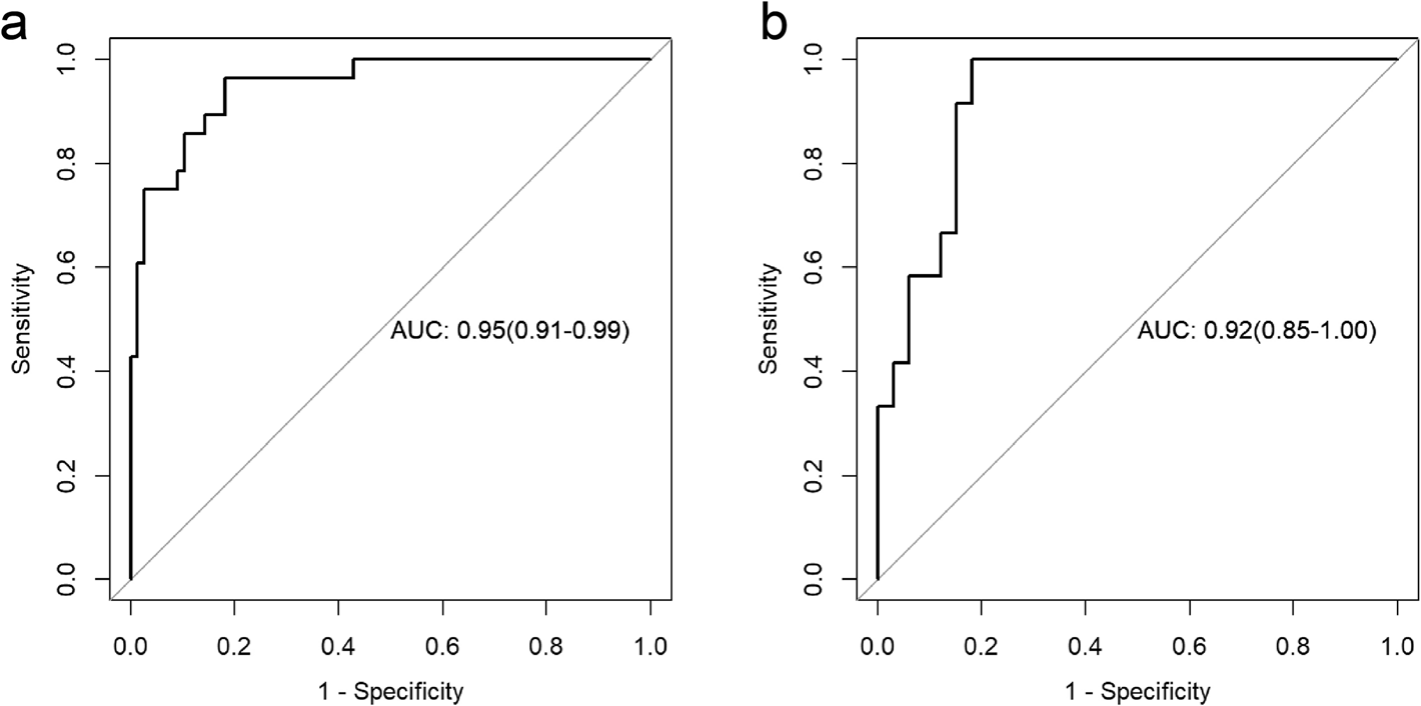
Receiver operating characteristic (ROC) curves of Radiomic features for the training (**a**) and test cohorts (**b**). The AUC for the training cohort and the test cohort was 0.95 and 0.92, respectively

As shown in Fig. 3, by LASSO regression analysis, the optimized hyperparameter λ was 0.00677, and 7 radiomic features remained, which included GLCMEntropy_AllDirection_offset1_SD, VoxelVolume, MajorAxisLength, RunLengthNonuniformity_AllDirection_offset7_SD, sumAverage, HaraEntroy and LongRunHighGreyLevelEmphasis_angle0_offset1. The formula of rad-score was as following:

Radscore = 1.46 × HaraEntroy+ 1.98 × LongRunHighGreyLevelEmphasis_Angle0_offset1 + 0.68*MajorAxisLength+ 0.761 × RunLengthNonuniformity_AllDirection_offset7_SD-0.38 × GLCMEntropy_AllDirection_offset1_SD + 0.89 × sumAverage-0.28 × VoxelVolume-1.82. The ROCs of the radiomics model in training cohort and test cohort are shown in Fig. 4. The AUCs were 0.95 (0.91–0.99) and 0.92(0.85–1.00) in training and test cohorts, respectively. The accuracy, sensitivity, specificity, PPV and NPV in the training cohort were 0.86, 0.96, 0.82, 0.66, 0.98, and were 0.87, 1.00, 0.82, 0.67, 1.00 in the test cohort.

### Construction and validation of the developed nomogram

**Fig. 5:**
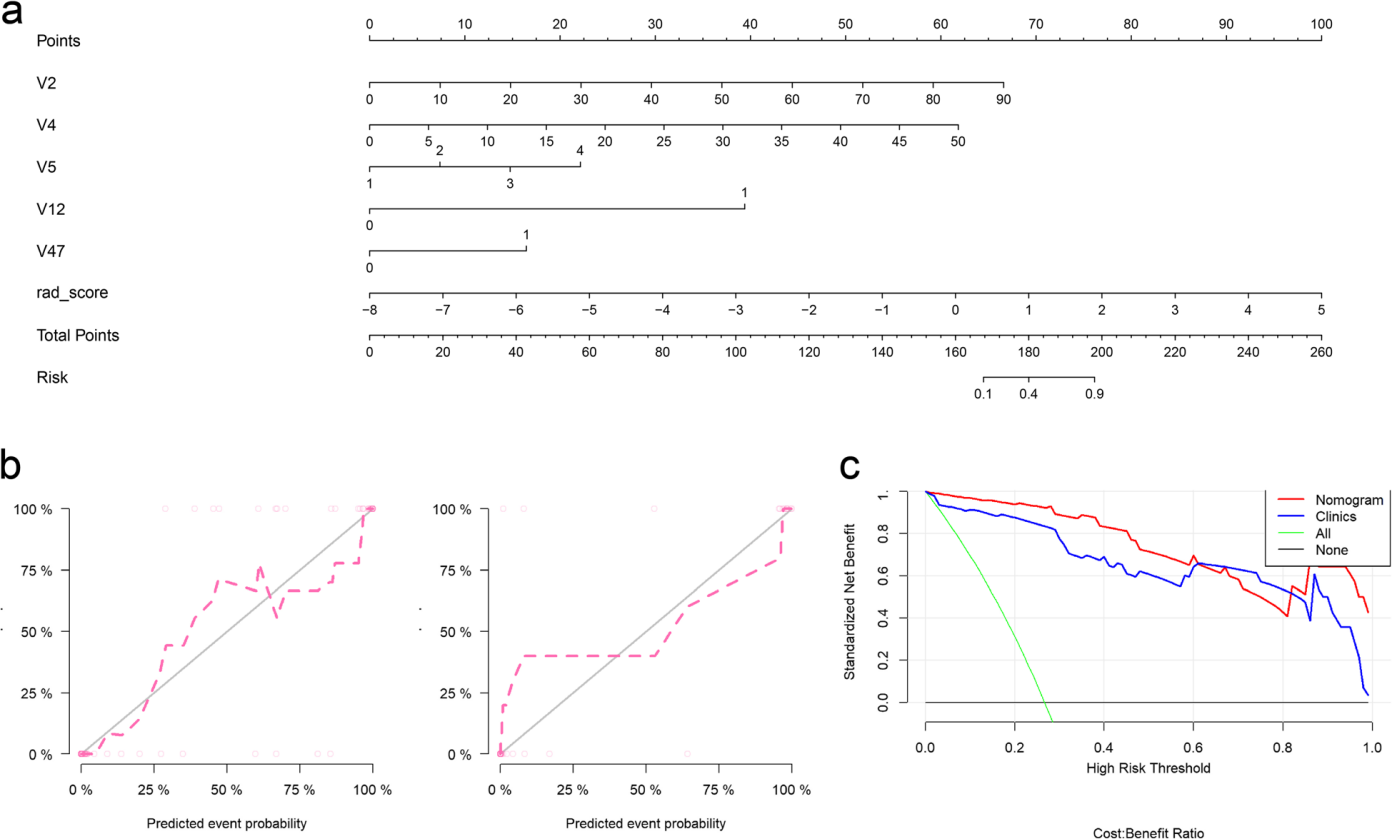
**a** Radiomics nomogram for identifying severity of COVID-19. **b** calibration curves of the radiomics nomogram in the training set and test cohort. The calibration curves represented calibration of the nomogram on the basis of fitting the predicted probabilities and observed probabilities. The 45° line uncovers the perfect discrimination and the dotted lines reveals the discriminative ability of the nomogram. The nearer the dotted line fits to the ideal line, the better the discriminative accuracy of the developed nomogram. **c** Decision-curve analysis for the radiomics nomogram. The y-axis and x-axis represent the net benefit and threshold probability, respectively. The horizontal black line indicates the assumption of all severe COVID-19 patients, while the green line indicates the assumption of all non-severe COVID-19 patients

The final statistically significant clinical features and Rad-score were used to construct the nomogram (Fig. 5a). The AUCs were 0.99 (0.98–1.00) and 0.98 (0.94–1.00) for the training and test cohorts, respectively. The performance of nomogram was shown as follows: the accuracy, sensitivity, specificity, PPV and NPV were 0.95, 1.00, 0.94, 0.85, 1.00 in the training cohort and were 0.89, 0.71, 1.00, 1.00, 0.85 in the test cohort. The calibration curve of the model in training and test cohorts and a non-statistical Hosmer-Lemeshow test (*P* > 0.05) both indicated well discrimination of the constructed nomogram (Fig. 5b). By decision curve analysis (Fig. 5c), with the risk thresholds ranged from 0 to 0.6 and 0.8 to 1, the nomogram represented higher net benefit in clinical practice.

## Discussion

In the present study, we developed and validated a predictive nomogram incorporating radiomics signatures and clinical data for further precisely discriminating the severity of COVID-19 patients. The results uncovered that the addition of radiomic characteristics to clinical model could get better performance in discriminating the severity of patients with COVID-19, with an elevated AUC (from 0.895 to 0.952) and a relatively higher sensitivity, specificity, PPV, and NPV in the test cohort. Moreover, the high NPV and specificity indicated that the developed model was reliable and could minimize the number of false-positive and false-negative patients, which is valuable in present clinical work [17]. The relatively high PPV implied that the clinical-radiomics model contributed to discriminating true high-risk patients. Based on our research, those high-risk patients could be recommended to receive more follow-up imaging to monitor changes in the condition. Furthermore, in this study, we constructed a clinical-radiomics nomogram as an individualized and visualized model to optimize the accuracy of assessing the severity of confirmed COVID-19 patients. The novel radiomics nomogram manifested favorable calibration and clinical benefit, verified by calibration curves and DCA.

As we know, radiomics has been widely applied in tumor research due to its merits of unwatched filter of comprehensive data obtained from an image. For example, radiomics could differentiate tumor heterogeneity and has often been performed to predict the prognosis of various cancers [18, 19]. Hence, radiomics, a noninvasive, fast, reproducible, and low-cost technic, was utilized in COVID-19 for identifying the severity, so as to avoid unnecessary treatment and decrease patients’ anxiety, especially when human and material resources are extremely precious. According to our experience, it took about 4–5 days to extract the radiomic features for all CT images and the model construction and validation only took 1 h. Once the model was established, we only needed to deploy the model in CT reading platform. Compared with the “traditional” CT reading, the radiologists simply needed to outline the lesion, and the machine would subsequently provide them with an objective result for their reference. Huang et al. [11] analyzed 154 viral pneumonia patients (including 89 cases of COVID-19 and 65 cases of influenza pneumonia) to establish a CT-based radiomics model, whose results showed radiomics model had a satisfactory performance in distinguishing COVID-19 and influenza pneumonia. Liu et al. [12] reported CT-based radiomics model could facilitate a rapid and accurate detection in differentiation of COVID-19 and Non-COVID-19 pneumonia. Fang et al. [13] summarized 136 patients with COVID-19 and developed a CT-based radiomics model for discriminating COVID-19 and other types of viral pneumonia, which showed a good performance for predicting COVID-19 pneumonia. Homayounieh et al. [20] summarized 315 patients with COVID-19 and developed a CT-based whole lung radiomics model, which showed a better performance in predicting outcome and disease severity of patients with COVID-19 compared with subjective assessment by radiologists.

Our study also showed that clinical data including age and comorbidity were associated with the severity of COVID-19. These results were in line with the facts that the elderly patients with other diseases are more likely to suffer from severe pneumonia, in accordance with the other studies [4]. It may be due to poor immune function of the elderly patients. Ruan et al. [3] verified that age and underlying diseases are predictors of a worse outcome in COVID-19. Wang et al. [21] also found elderly age complicated with underlying diseases might serve as important risk factors for the severity of COVID-19. However, several researchers found that multiple laboratory indicators may be linked with the severity of patients [22]. Cytokine storm, comorbid various infections and inhibited immune function may lead to increased ratio of neutrophil, decreased lymphocytes, elevated C-reactive protein and procalcitonin. However, these were not significant factors in the present study. The possible reasons may be the differences in sample size and statistical methods. In addition, radiological findings, including CT scores, number of lesions and GGO with consolidation were also independent indicators of the severity of COVID-19. Compared with non-severe cases, severe patients were more likely to involve a wider range of both lungs, signifying more lesions and higher CT scores (number of lung segments involved). Several studies suggested that as the course of the disease increases, lesions in the lungs increase and worsen [23], which is also in line with our results. Moreover, GGO with consolidation appeared more frequent in severe/critical patients, implying that the alveoli damage is more filled by inflammatory exudation, such as fibromyxoid exudates [24]. The rest of CT findings in our study were not significantly different between severe and non-severe groups.

Our study has some limitations. First, this retrospective study posed inevitable selection bias. Second, ROI delineation was manual, and the irregularities of lesions might cause differences in the manual selection. In addition, the study used 2D ROI selection due to time and technical constraints, yet 3D ROI selection should be used for further research in the future. The sample size in our cohort was relatively small. Third, the relationship between prognosis and clinical-radiological characteristics has not been studied. Thus, further studies with more cases and prolonged period should be studied to further verify our results.

In conclusion, we introduced a CT-based radiomics nomogram to evaluate the feasibility of radiomics signature and clinical factors in discriminating the severity of COVID-19 patients. Besides, we developed and validated the radiomics nomogram incorporating radiomics signatures, age, comorbidity, CT scores, number of lesions and GGO with consolidation, which improved the diagnostic performance in severity stratification of COVID-19 patients.

## Data Availability

The datasets used and/or analyzed during the current study are available from the corresponding author on reasonable request.

## Abbreviations

AUC: Area Under the Curve
CI: Confidence interval
COVID-19: Coronavirus disease 2019
CT: Computed tomography
DCA: Decision curve analysis
GGO: Ground ground-glass opacity
HU: Hounsfield unit
ICC: Intraclass correlation coefficient
LASSO: Least absolute shrinkage selection operator
mRMR: Max-Relevance and Min-Redundancy
NPV: Negative predictive value
OR: Odds ratio
RT-PCR: Real-time reverse transcriptase polymerase chain reaction
ROC: Receiver operating characteristic
PPV: Positive predictive value; ROI: regions of interest.
